# Supplementary material for: Generation and comparison of CRISPR-Cas9 and Cre-mediated genetically engineered mouse models of sarcoma
Source: Nat Commun. 2017 Jul 10;8:15999. doi: 10.1038/ncomms15999 (PMC5508130; doi:10.1038/ncomms15999)
Supplement: Supplementary Information [file ncomms15999-s1.pdf]

File name: Supplementary Information
Description: Supplementary figures and supplementary tables.

File name: Peer review file
Description:

Supplementary Figure 1

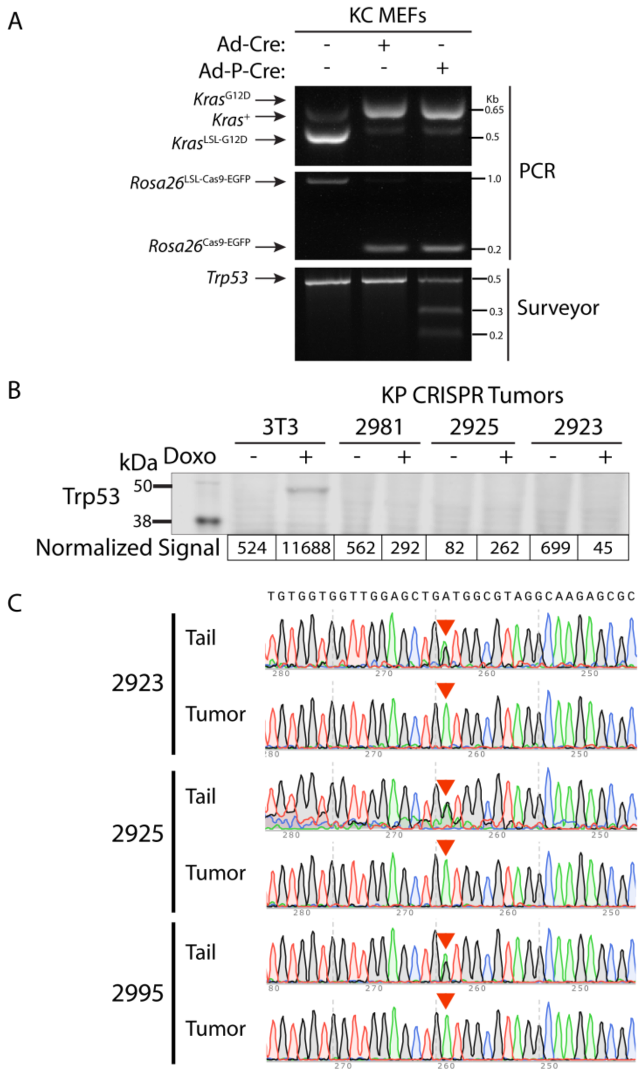

Supplementary Figure 1. Validation of Ad-sgTrp53-Cre adenovirus particles and *Kras* and *Trp53* status in KP CRISPR tumors. (A) PCR analysis of genomic DNA from KC MEFs following Ad-sgTrp53-Cre transduction demonstrates successful Cre-mediated recombination of loxP-STOP-loxP cassettes as shown by the presence of the *Kras*^{G12D} and *Rosa26*$^{Cas9\text{-}EGFP}$ bands (and corresponding absence of *Kras*$^{LSL\text{-}G12D}$ and *Rosa26*$^{LSL\text{-}Cas9\text{-}EGFP}$ bands) in KC MEFs transduced with Ad-Cre or Ad-sgTrp53-Cre. Surveyor assay shows the presence of cleaved heteroduplexes only in Ad-sgTrp53-Cre transduced KC MEFs confirming sgTrp53 and Cas9 activity. (B) Western blot for Trp53 shows that Trp53 expression is not induced in CRISPR-generated KP tumors after exposure to 0.5 µg/mL doxorubicin for 8 hours. 3T3 mouse fibroblasts served as a positive control for induction of Trp53 expression. Target protein quantification was normalized to total protein. (C) Detection of wild type Kras and Kras^{G12D} alleles. Both wild type Kras (G, black) and Kras^{G12D} (A, green) alleles were detected in KC tails, but only Kras^{G12D} was detected in KC tumors. The result is representative of at least two different experiments.

Supplementary Figure 2

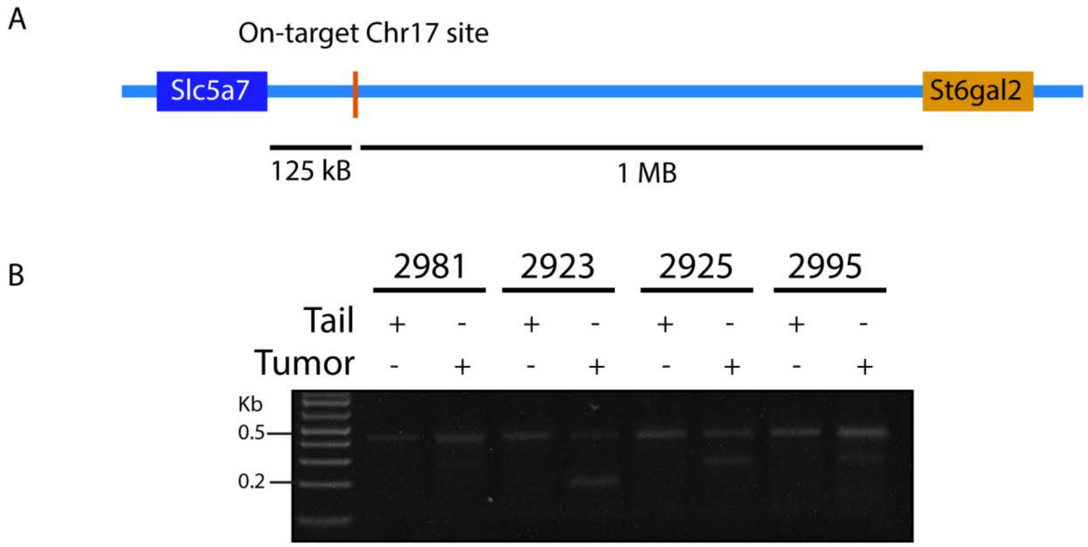

Supplementary Figure 2. Validation of sgTrp53 on-target chromosome 17 site. (A) Nearest adjacent protein coding genes to the sgTrp53 targeted region on chromosome 17. (B) Surveyor analysis revealed indels in the on-target chromosome 17 site in the same four cell lines from which we analyzed *Trp53* modification.

Supplementary Figure 3

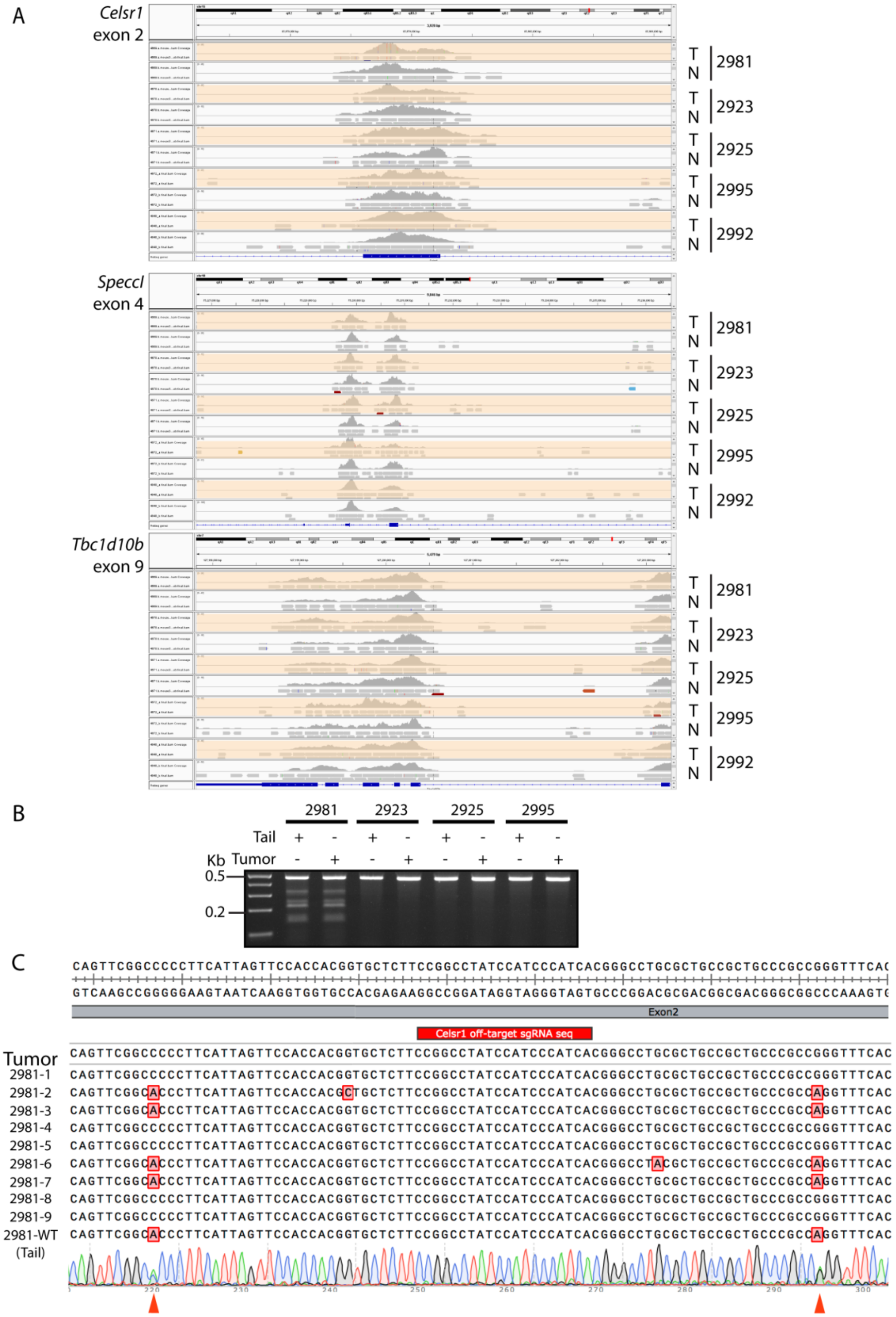

Supplementary Figure 3. *Celsr1* off-target validation. (A) Surveyor analysis of off-target *Celsr1* exon 2 in the same four cell lines from which we analyzed *Trp53* modification. (B) Sanger sequencing of off-target *Celsr1* exon 2 in the tumor cell lines-2981 with the same mouse tail DNA as control. T = tumor, N = normal.

Supplementary Figure 4

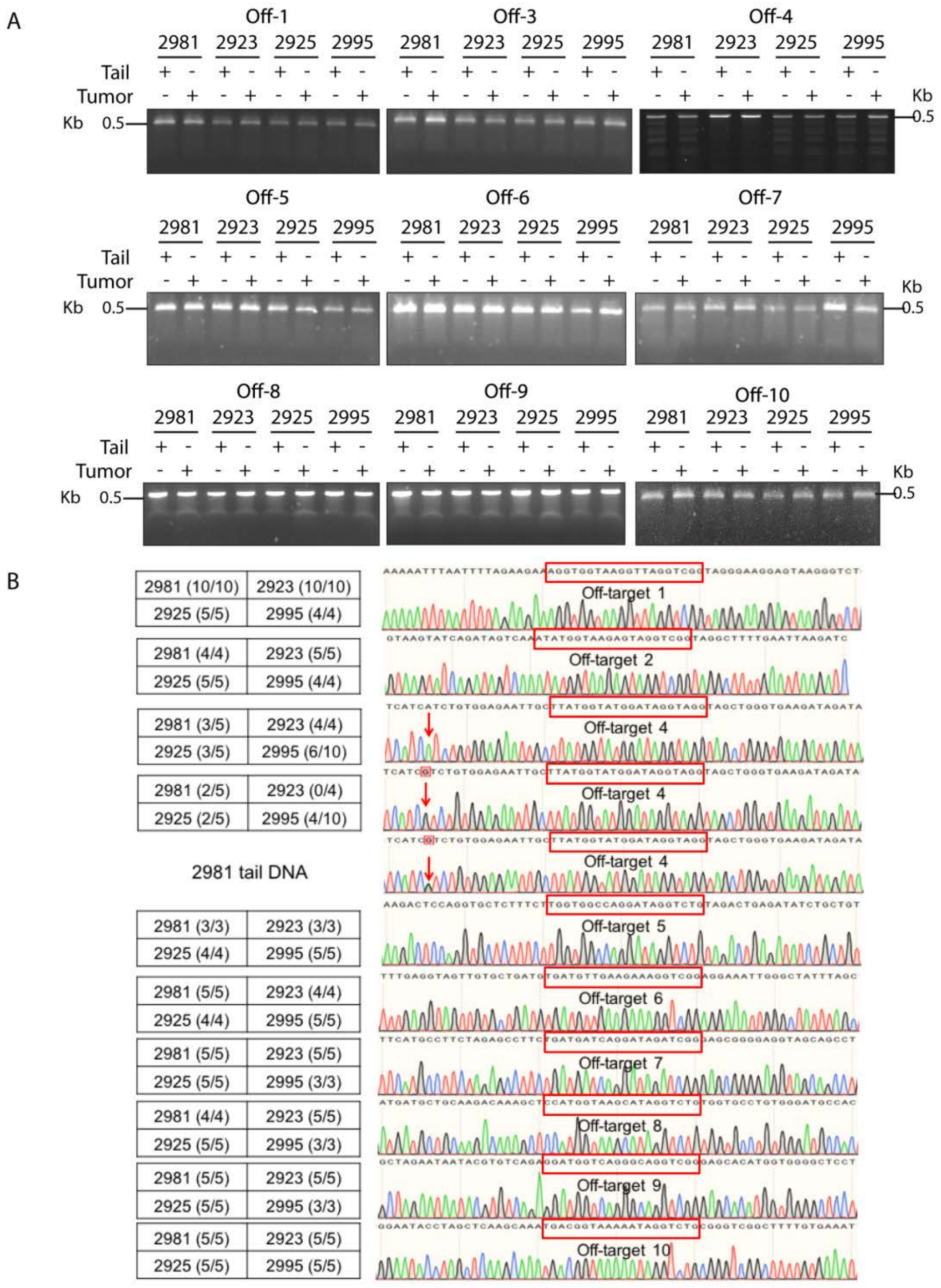

Supplementary Figure 4. Validation of the remaining top 9 off-targets. (A) Surveyor analysis of remaining top 9 off-targets in the same four cell lines from which we analyzed *Trp53* modifications. (B) Sanger sequencing of remaining top 9 off-targets in the same four cell lines from which we analyzed *Trp53* modifications.

Supplementary Figure 5

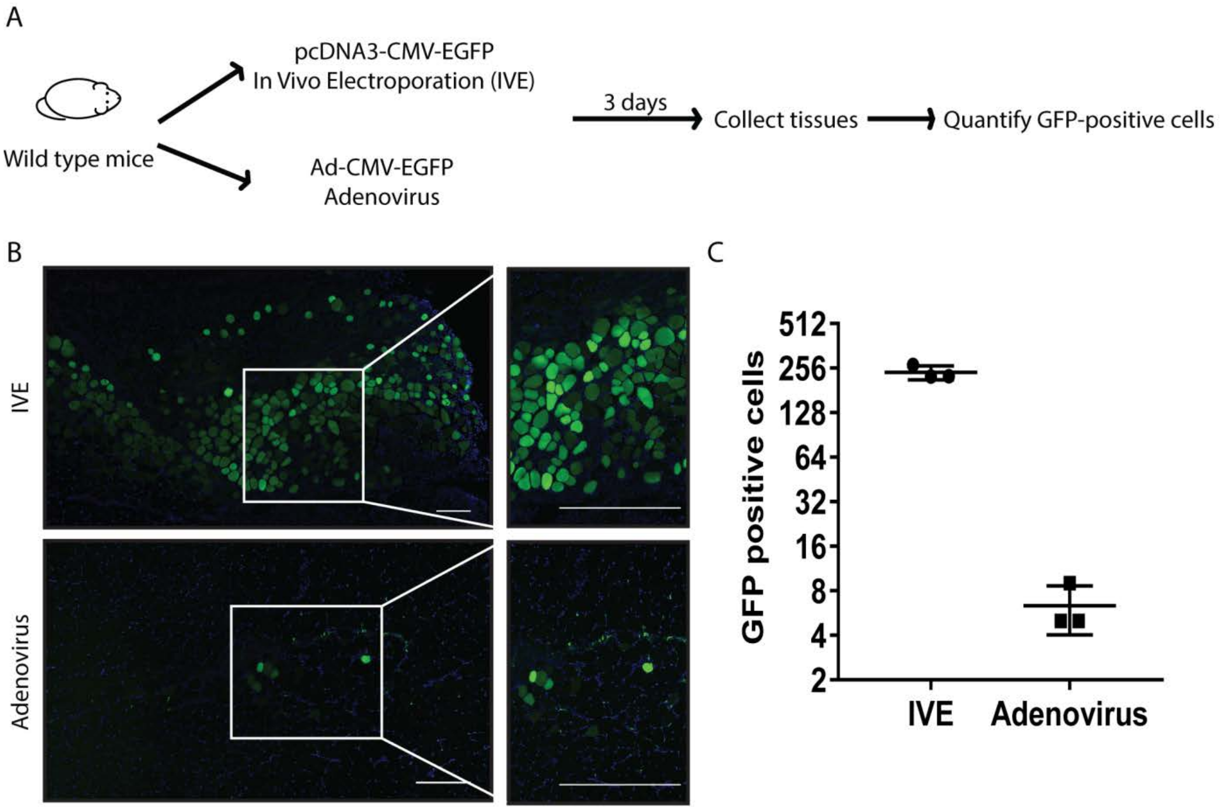

Supplementary Figure 5. **Comparison of the efficiency of GFP expression in muscle after in vivo electroporation (IVE) and adenovirus delivery.** (A) Wild type mice received intramuscular delivery of either an adenovirus expressing EGFP (n = 3) or naked plasmid pcDNA3-EGFP with IVE (n = 3). Skeletal muscle from the site of injection or IVE was analyzed for GFP expression 3 days later. (B) Fluorescence microscopy was performed and representative images are shown. (C) Quantification of the number of GFP-positive cells in skeletal muscle in a section from three different mice that received intramuscular injection of adenovirus or IVE. Scale bars = 200 µm.

Supplementary Figure 6

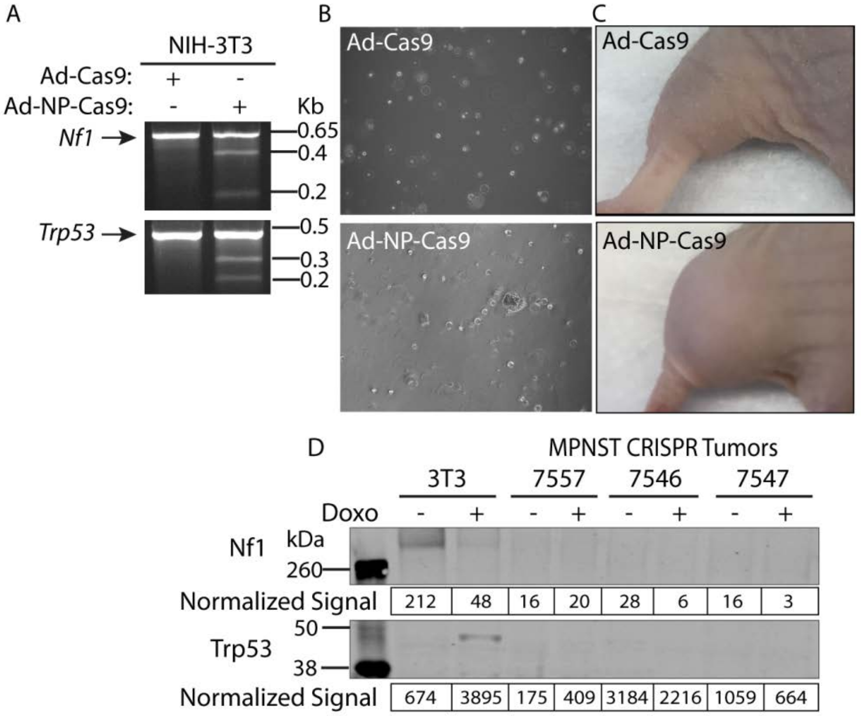

Supplementary Figure 6. Validation of Ad-sgNf1-sgTrp53-Cas9 adenovirus particles *in vitro* and *in vivo*. (A) Surveyor assay detects indels by the presence of cleaved heteroduplexes in genomic DNA isolated from 3T3 cells transduced with Ad-sgNf1-sgTrp53-Cas9. No indels were detected when Ad-Cas9 was introduced with no sgRNAs targeting *Nf1* or *Trp53*. (B) Wild type MEFs transduced with Ad-sgNf1-sgTrp53-Cas9 are transformed and formed colonies in soft agar, but MEFs transduced with Ad-Cas9 alone are not transformed and do not form colonies. The result is representative of at least two different experiments. (C) MEFs transformed with Ad-sgNf1-sgTrp53-Cas9 form tumors *in vivo* after allotransplantation into nude mice (*n* = 2). (D) Western blot

Supplementary Figure 7

Supplementary Figure 1B.

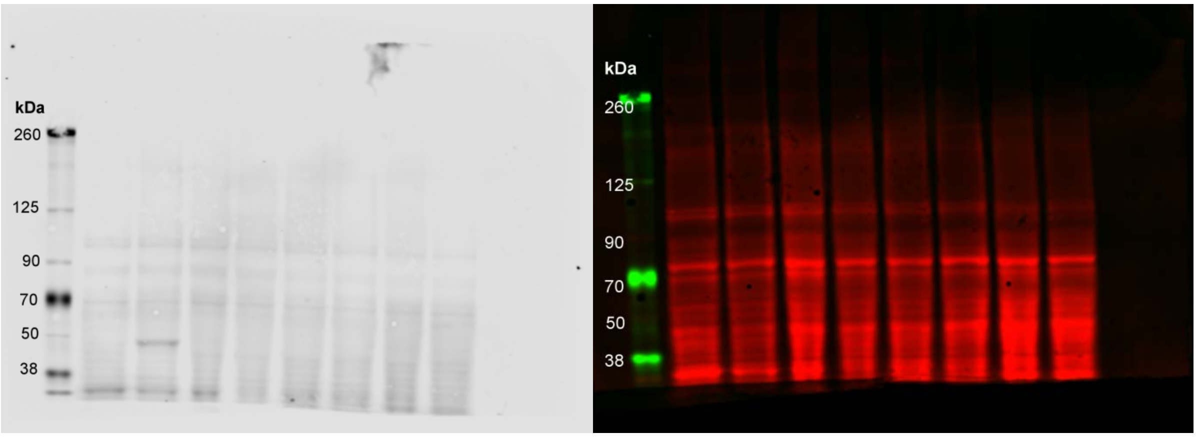

Supplementary Figure 6D.

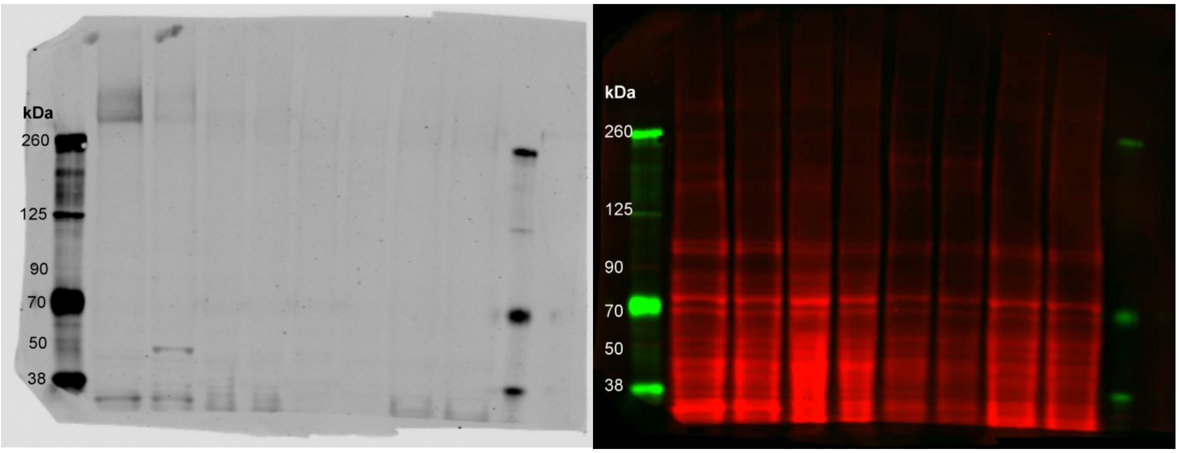

Supplementary Figure 7. Images of full western blots. Immunoblotting, image acquisition and analysis parameters are detailed in the methods.

Supplementary Table 1

sgRNA sequences (PAM sequence in bold)

Target gene	sgRNA ID	Target sequence (5' to 3')
Trp53	sgTrp53	GATGGTAAGGATAGGTCGG**CGG**
Nf1	sgNf1	GGACATCTCCAAGGATGTGG**TGG**

Supplementary Table 2

Detailed summary of deep sequencing of 2 on-target sequences

Normal muscles						Tumors					
#	% Indels	Total Reads	Deletion	Insertion	Mismatch	#	% Indels	Total Reads	Deletion	Insertion	Mismatch
On-target *Trp53* #2981	0.03%	65030	0.03%	0.00%	2.17%	On-target *Trp53* #2981	0.74%	36123	0.50%	0.24%	2.12%
On-target *Trp53* #2995	0.05%	36472	0.05%	0.00%	2.24%	On-target *Trp53* #2995	27.87%	47553	27.87%	0.00%	2.51%
On-target *Trp53* #2925	0.05%	48063	0.05%	0.00%	2.00%	On-target *Trp53* #2925	52.30%	36831	52.30%	0.00%	2.77%
On-target *Trp53* #2923	0.05%	49966	0.05%	0.00%	2.15%	On-target *Trp53* #2923	0.04%	45591	0.04%	0.00%	2.21%
On-target *Trp53* #2992	0.04%	44945	0.04%	0.00%	2.08%	On-target *Trp53* #2992	1.02%	51076	0.80%	0.22%	2.09%
On-target Chr17 #2981	0.39%	4E+05	0.34%	0.04%	3.52%	On-target Chr17 #2981	41.18%	246157	40.99%	0.18%	3.93%
On-target Chr17 #2995	0.37%	3E+05	0.32%	0.06%	3.48%	On-target Chr17 #2995	16.92%	243294	10.92%	6.00%	4.48%
On-target Chr17 #2925	0.37%	4E+05	0.33%	0.04%	3.42%	On-target Chr17 #2925	57.82%	280932	57.01%	0.81%	2.02%
On-target Chr17 #2923	0.34%	3E+05	0.31%	0.03%	3.15%	On-target Chr17 #2923	10.57%	397631	0.36%	10.21%	3.76%
On-target Chr17 #2992	0.45%	4E+05	0.33%	0.12%	3.38%	On-target Chr17 #2992	80.70%	73647	80.68%	0.02%	3.31%

Supplementary Table 3

Detailed summary of deep sequencing of top 10 off-target sequences

**Normal muscles**						**Tumors**					
**#**	**%Indels**	**Total Reads**	**Deletion**	**Insertion**	**Mismatch**	**#**	**%Indels**	**Total Reads**	**Deletion**	**Insertion**	**Mismatch**
OFF1 #2981	0.05%	53433	0.05%	0.00%	4.47%	OFF1 #2981	0.05%	42036	0.05%	0.00%	4.50%
OFF1 #2995	0.06%	44163	0.05%	0.00%	4.74%	OFF1 #2995	0.07%	64554	0.07%	0.00%	4.52%
OFF1 #2925	0.05%	56572	0.05%	0.00%	4.41%	OFF1 #2925	0.05%	48235	0.05%	0.00%	4.32%
OFF1 #2923	0.06%	54952	0.06%	0.00%	4.59%	OFF1 #2923	0.04%	39087	0.04%	0.00%	4.41%
OFF1 #2992	0.04%	54341	0.04%	0.00%	4.04%	OFF1 #2992	0.07%	52933	0.07%	0.00%	4.54%
OFF2 #2981	0.03%	46370	0.03%	0.00%	2.49%	OFF2 #2981	0.04%	38939	0.04%	0.00%	2.33%
OFF2 #2995	0.05%	38401	0.05%	0.00%	2.33%	OFF2 #2995	0.05%	57868	0.05%	0.00%	2.17%
OFF2 #2925	0.04%	71547	0.04%	0.00%	2.19%	OFF2 #2925	0.03%	53459	0.03%	0.00%	2.12%
OFF2 #2923	0.03%	46992	0.03%	0.00%	2.19%	OFF2 #2923	0.04%	38201	0.04%	0.00%	2.15%
OFF2 #2992	0.03%	53927	0.03%	0.00%	2.29%	OFF2 #2992	0.05%	48577	0.05%	0.00%	2.22%
OFF3 #2981	0.04%	54319	0.04%	0.00%	2.23%	OFF3 #2981	0.02%	46119	0.02%	0.00%	2.16%
OFF3 #2995	0.03%	39117	0.03%	0.00%	2.09%	OFF3 #2995	0.03%	48703	0.03%	0.00%	2.15%
OFF3 #2925	0.02%	54653	0.02%	0.00%	2.21%	OFF3 #2925	0.03%	48774	0.03%	0.00%	2.09%
OFF3 #2923	0.03%	50452	0.03%	0.00%	2.13%	OFF3 #2923	0.02%	39669	0.02%	0.00%	1.96%
OFF3 #2992	0.03%	53132	0.03%	0.00%	2.01%	OFF3 #2992	0.03%	46071	0.03%	0.00%	2.00%
OFF4 #2981	0.03%	47392	0.03%	0.00%	3.26%	OFF4 #2981	0.02%	37063	0.02%	0.00%	3.72%

OFF4 #2995	0.05%	43544	0.05%	0.00%	2.24%	OFF4 #2995	0.03%	67187	0.03%	0.00%	3.36%
OFF4 #2925	0.04%	61562	0.04%	0.00%	3.38%	OFF4 #2925	0.02%	50434	0.02%	0.00%	3.34%
OFF4 #2923	0.04%	52788	0.04%	0.00%	3.25%	OFF4 #2923	0.02%	47469	0.02%	0.00%	3.02%
OFF4 #2992	0.07%	53182	0.07%	0.00%	2.74%	OFF4 #2992	0.03%	60838	0.03%	0.00%	3.14%
OFF5 #2981	0.03%	64550	0.03%	0.00%	2.27%	OFF5 #2981	0.02%	56234	0.02%	0.00%	2.07%
OFF5 #2995	0.02%	56273	0.02%	0.00%	2.24%	OFF5 #2995	0.01%	75993	0.01%	0.00%	2.07%
OFF5 #2925	0.02%	87744	0.02%	0.00%	2.16%	OFF5 #2925	0.02%	68166	0.02%	0.00%	2.02%
OFF5 #2923	0.02%	68971	0.02%	0.00%	2.06%	OFF5 #2923	0.03%	56517	0.03%	0.00%	2.20%
OFF5 #2992	0.03%	62091	0.03%	0.00%	2.14%	OFF5 #2992	0.03%	71476	0.03%	0.00%	1.99%
OFF6 #2981	0.03%	67794	0.03%	0.00%	2.55%	OFF6 #2981	0.05%	60207	0.05%	0.00%	2.40%
OFF6 #2995	0.02%	52935	0.02%	0.00%	2.76%	OFF6 #2995	0.05%	95712	0.05%	0.00%	2.49%
OFF6 #2925	0.05%	79583	0.05%	0.00%	2.54%	OFF6 #2925	0.05%	62360	0.05%	0.00%	2.33%
OFF6 #2923	0.05%	63569	0.05%	0.00%	2.50%	OFF6 #2923	0.04%	52736	0.04%	0.00%	2.46%
OFF6 #2992	0.05%	65810	0.05%	0.00%	2.46%	OFF6 #2992	0.04%	81825	0.04%	0.00%	2.29%
OFF7 #2981	0.08%	57832	0.08%	0.00%	2.25%	OFF7 #2981	0.06%	53987	0.06%	0.00%	2.08%
OFF7 #2995	0.06%	50877	0.06%	0.00%	2.54%	OFF7 #2995	0.07%	49535	0.07%	0.00%	2.30%
OFF7 #2925	0.04%	57497	0.04%	0.00%	2.28%	OFF7 #2925	0.06%	51985	0.06%	0.00%	2.15%
OFF7 #2923	0.05%	57676	0.05%	0.00%	2.18%	OFF7 #2923	0.06%	47201	0.06%	0.00%	2.23%
OFF7 #2992	0.08%	57618	0.08%	0.00%	2.17%	OFF7 #2992	0.05%	51688	0.05%	0.00%	2.16%
OFF8 #2981	0.04%	52189	0.04%	0.00%	2.36%	OFF8 #2981	0.02%	35691	0.02%	0.00%	2.18%

OFF8 #2995	0.03%	4308 2	0.03%	0.00%	2.42%	OFF8 #2995	0.04%	5038 2	0.04%	0.00%	2.29%
OFF8 #2925	0.03%	6381 5	0.03%	0.00%	2.32%	OFF8 #2925	0.03%	5667 4	0.03%	0.00%	2.18%
OFF8 #2923	0.03%	5757 3	0.03%	0.00%	2.30%	OFF8 #2923	0.02%	4108 7	0.02%	0.00%	2.12%
OFF8 #2992	0.02%	6415 5	0.02%	0.00%	2.44%	OFF8 #2992	0.05%	5548 0	0.05%	0.00%	2.12%
OFF9 #2981	0.04%	5965 4	0.04%	0.00%	2.09%	OFF9 #2981	0.03%	5675 4	0.03%	0.00%	2.12%
OFF9 #2995	0.03%	5057 5	0.03%	0.00%	2.17%	OFF9 #2995	0.02%	6633 8	0.02%	0.00%	2.06%
OFF9 #2925	0.03%	6684 2	0.03%	0.00%	2.27%	OFF9 #2925	0.03%	6798 7	0.03%	0.00%	2.03%
OFF9 #2923	0.03%	6270 1	0.03%	0.00%	2.12%	OFF9 #2923	0.03%	7044 5	0.03%	0.00%	2.11%
OFF9 #2992	0.02%	5938 4	0.02%	0.00%	1.97%	OFF9 #2992	0.02%	6536 7	0.02%	0.00%	1.99%
OFF1 0 #2981	0.09%	7211 8	0.09%	0.00%	1.81%	OFF1 0 #2981	0.09%	8694 8	0.09%	0.00%	1.70%
OFF1 0 #2995	0.08%	5942 6	0.08%	0.00%	1.79%	OFF1 0 #2995	0.09%	8208 6	0.09%	0.00%	1.69%
OFF1 0 #2925	0.09%	7234 0	0.09%	0.00%	1.68%	OFF1 0 #2925	0.07%	7176 1	0.07%	0.00%	1.70%
OFF1 0 #2923	0.08%	7488 5	0.08%	0.00%	1.76%	OFF1 0 #2923	0.10%	7287 3	0.10%	0.00%	1.74%
OFF1 0 #2992	0.07%	7065 5	0.07%	0.00%	1.84%	OFF1 0 #2992	0.07%	7847 1	0.07%	0.00%	1.59%

Supplementary Table 4

Predicted top 3 off-target exons and top 10 off-targets for sgTrp53 sequences (PAM sequence in bold)

Target gene	Target exon	sgRNA ID	Target sequence (5' to 3')
Celsr1	Exon 2	OFF-target exon 1	TGATGGGATGGATAGGCCGG**AAG**
Specc11	Exon 4	OFF-target exon 2	TGAAGGTGAGGACAGGTCTG**AGG**
Tbc1d10b	Exon 9	OFF-target exon 3	TGATGGTGGGGACAGGTCAG**CAG**
		OFF-target 1	AGGTGGTAAGGTTAGGTCGG**TAG**
		OFF-target 2	TGATGGGATGGATAGGCCGG**AAG**
		OFF-target 3	ATATGGTAAGAGTAGGTCGG**TAG**
		OFF-target 4	TTATGGTATGGATAGGTAGG**TAG**
		OFF-target 5	TGGTGGCCAGGATAGGTCTG**TAG**
		OFF-target 6	TGATGTTGAAGAAAGGTCGG**AGG**
		OFF-target 7	TGATGATCAGGATAGATCGG**GAG**
		OFF-target 8	CCATGGTAAGCATAGGTCTG**TGG**
		OFF-target 9	GGATGGTCAGGGCAGGTCGG**GAG**
		OFF-target 10	TGACGGTAAAAATAGGTCTG**CGG**

Supplementary Table 5

Primer sequences

Primer ID	Sequence (5' to 3')	Use
sgTrp53 5.1	CACCGGATGGTAAGGATAGGTCGG	Cloning sgTrp53
sgTrp53 3.1	AAACCCGACCTATCCTTACCATCC	
sgTrp53 svg 5.1	TGCCGAACAGGTGGAATATC	Amplify Trp53 locus for Surveyor + TOPO cloning
sgTrp53 svg 3.1	CATCCTGACTGTGTGTAAACTAGGCCCC	
sgNf1 5.1	CACCGGGACATCTCCAAGGATGTGG	Cloning sgNf1
sgNf1 3.1	AAACCCACATCCTTGGAGATGTCCC	
sgNf1 svg 5.1	TAGCAATTTTGGGGGAACGC	Amplify Nf1 locus for Surveyor
sgNf1 svg 3.1	GTCAGAGCCTTTCAGTCATG	
LSL-Cas9 genotyping 5.1	CTTTTTCCTACAGCTCCTGG	Genotyping for recombination of Cas9 allele
LSL-Cas9 genotyping 3.1	CATGGTGGCACGTGTTCGAA	
mt KrasG12D 5.1	TGTAAGGCCTGCTGAAAATGACT	Detection of WT Kras and $Kras^{G12D}$ allele
mt KrasG12D 3.1	TGGACCTTACTCTCTGTGGT	
sgTrp53 chr17 on-target svg 5.1	ATCTCAAAGCGTTCACGCCC	Amplify on target chr17 site for Surveyor + TOPO cloning
sgTrp53 chr17 on-target svg 3.1	CGAATATCTGGAAGACAGGC	
Celsr1 svg 5.1	ATCCAGAACGACACGGACGT	Amplify on off-target top2 Celsr1 exon2 for Surveyor + TOPO cloning
Celsr1 svg 3.1	CACAGGCTCCAGACACTTAC	
Off-1 svg 5.1	GTGGATAGAGACTGCTTTGG	Amplify on off-target 1 for Surveyor + TOPO cloning
Off-1 svg 3.1	GGGTGTGTTCCCAGAATTCA	
Off-3 svg 5.1	GGAATGTGCCCATCTGCAAA	Amplify on off-target 3 for Surveyor + TOPO cloning
Off-3 svg 3.1	GAAGTCCTTCCTGATCCTCC	
Off-4 svg 5.1	ATGGCATCCACTCTGCTCTT	Amplify on off-target 4 for Surveyor + TOPO cloning
Off-4 svg 3.1	AGTCATAAGCAGCCCCTCTG	
Off-5 svg 5.1	GAGTGTCCTCAAGCAGTTTG	Amplify on off-target 5

Off-5 svg 3.1	TCCAGGTACCAAACACACAG	for Surveyor + TOPO cloning
Off-6 svg 5.1	ACCACCAACAGGAGAACTTC	Amplify on off-target 6 for Surveyor + TOPO cloning
Off-6 svg 3.1	TATTTGCAGTCACGCTGGGC	
Off-7 svg 5.1	GCCCCATGAAGCCTCAGTTT	Amplify on off-target 7 for Surveyor + TOPO cloning
Off-7 svg 3.1	ACAAACCATGGGGACTTCCA	
Off-8 svg 5.1	TGAGTTCACAGATGCTTTCC	Amplify on off-target 8 for Surveyor + TOPO cloning
Off-8 svg 3.1	ATCGTGAAGCTGAGGTAGAA	
Off-9 svg 5.1	GTGTGCTCCAGCAAAACACC	Amplify on off-target 9 for Surveyor + TOPO cloning
Off-9 svg 3.1	TTCCGTCTATACTCCCCTCC	
Off-10 svg 5.1	ATGAACATGCTGCAGGCGAC	Amplify on off-target 10 for Surveyor + TOPO cloning
Off-10 svg 3.1	ACCCAATCCCAGGCATTAAT	
ON-target Trp53 Fwd	TCGTCGGCAGCGTCAGATGTGTATAAGAGACAGGT AGGGAGCGACTTCACCTG	First round PCR for ON-target
ON-target Trp53 Rev	GTCTCGTGGGCTCGGAGATGTGTATAAGAGACAGC GCCTTCCTACCTGGAGTCT	
ON-target Chr17 Fwd	TCGTCGGCAGCGTCAGATGTGTATAAGAGACAGGC ACAAACACGAACCTCAAA	First round PCR for ON-target Chr17
ON-target Chr17 Rev	GTCTCGTGGGCTCGGAGATGTGTATAAGAGACAGC GGCTCTGAGTATACCACCA	
OFF-target1 Fwd	TCGTCGGCAGCGTCAGATGTGTATAAGAGACAGTG ATGGCACAGGGCCTTTAA	First round PCR for OFF-target1
OFF-target1 Rev	GTCTCGTGGGCTCGGAGATGTGTATAAGAGACAGG GTCATACTTATCCTTGCCT	
OFF-target2 Fwd	TCGTCGGCAGCGTCAGATGTGTATAAGAGACAGGC ACGGATTGGAGTAGCAAA	First round PCR for OFF-target2
OFF-target2 Rev	GTCTCGTGGGCTCGGAGATGTGTATAAGAGACAGT GAAATGCGTGTCTGTGCTT	
OFF-target3 Fwd	TCGTCGGCAGCGTCAGATGTGTATAAGAGACAGGC CTTCCGATGCTCTAATGA	First round PCR for OFF-target3
OFF-target3 Rev	GTCTCGTGGGCTCGGAGATGTGTATAAGAGACAGG CTGTTAATTTGAACACACCAAA	

OFF-target4 Fwd	TCGTCGGCAGCGTCAGATGTGTATAAGAGACAGCA GCTCTGGGCAATTTGTTT	First round PCR for OFF-target4
OFF-target4 Rev	GTCTCGTGGGCTCGGAGATGTGTATAAGAGACAGC TTGGCTTTCCAGTCCACAT	
OFF-target5 Fwd	TCGTCGGCAGCGTCAGATGTGTATAAGAGACAGGC TGTGGAAGACAGGGACTC	First round PCR for OFF-target5
OFF-target5 Rev	GTCTCGTGGGCTCGGAGATGTGTATAAGAGACAGT GTGGGGATGTGGAATTGTA	
OFF-target6 Fwd	TCGTCGGCAGCGTCAGATGTGTATAAGAGACAGGC CCAGTGGTGTGGTTAAAT	First round PCR for OFF-target6
OFF-target6 Rev	GTCTCGTGGGCTCGGAGATGTGTATAAGAGACAGA CTTGGAAACCGAGACCTCA	
OFF-target7 Fwd	TCGTCGGCAGCGTCAGATGTGTATAAGAGACAGAC AATGTGCTGACCCTGGAC	First round PCR for OFF-target7
OFF-target7 Rev	GTCTCGTGGGCTCGGAGATGTGTATAAGAGACAGG AGACAGCTGGGGTAACTGC	
OFF-target8 Fwd	TCGTCGGCAGCGTCAGATGTGTATAAGAGACAGCC ATGATTGTGCCTATGTGC	First round PCR for OFF-target8
OFF-target8 Rev	GTCTCGTGGGCTCGGAGATGTGTATAAGAGACAGC CATGTCCAGGATGGACTCT	
OFF-target9 Fwd	TCGTCGGCAGCGTCAGATGTGTATAAGAGACAGGG TCTACCTACTTCCACATTGC	First round PCR for OFF-target9
OFF-target9 Rev	GTCTCGTGGGCTCGGAGATGTGTATAAGAGACAGG GAGTTTTGTCACAGCCACA	
OFF-target10 Fwd	TCGTCGGCAGCGTCAGATGTGTATAAGAGACAGTG ATTTCAATGTGGCCTTCA	First round PCR for OFF-target10
OFF-target10 Rev	GTCTCGTGGGCTCGGAGATGTGTATAAGAGACAGA AGAAGAATGCCCCCTGATT	
Universal i5 primer	AATGATACGGCGACCACCGAGATCTACACTCGTCG GCAGCGTC	Second round PCR for MiSeq analysis
BC1	CAAGCAGAAGACGGCATACGAGATACATCGGTCTC GTGGGCTCGG	
BC2	CAAGCAGAAGACGGCATACGAGATTGGTCAGTCTC GTGGGCTCGG	
BC3	CAAGCAGAAGACGGCATACGAGATCACTGTGTCTC GTGGGCTCGG	

BC4	CAAGCAGAAGACGGCATACGAGATATTGGCGTCTCGTGGGCTCGG	
BC5	CAAGCAGAAGACGGCATACGAGATGATCTGGTCTCGTGGGCTCGG	
BC6	CAAGCAGAAGACGGCATACGAGATTACAAGGTCTCGTGGGCTCGG	
BC7	CAAGCAGAAGACGGCATACGAGATCGTGATGTCTCGTGGGCTCGG	
BC8	CAAGCAGAAGACGGCATACGAGATGCCTAAGTCTCGTGGGCTCGG	
BC9	CAAGCAGAAGACGGCATACGAGATTCAAGTGTCTCGTGGGCTCGG	
BC10	CAAGCAGAAGACGGCATACGAGATAGCTAGGTCTCGTGGGCTCGG	

Supplementary Table 6

Lists of validated mutations in each tumor tissue

Method	Tumor	Validated mutation variants				
CreLox	4014	Ttn	Limch1	Cdc27		
	4015	Rnf152	C3ar1	St6galnac2		
	4017					
	4018	Secisbp2l	Col25a1	Grm8	Otog	Vmn2r79
		Ces1c	Trpc1	Naca	Fat2	Drd3
		Cldn8				
	4019					
	4020	Golga3	Myom1			
	4021					
CRISPR-Cas9-Cre-Lox	2923					
	2925	Met	Hipk2	Tekt1	Tsen54	Clybl
		Tssk1	Mep1a			
	2995	Olfr376	Mycs			
	2992	Olfr644	Adgra3	Nlrp1b	Smc3	
	2981					
